# Investigation of Long Non-Coding RNAs *H19* rs3741219, *MEG3* rs7158663, *POLR2E* rs3787016, and *ANRIL* rs10757274 with Breast Cancer Susceptibility and Clinicopathological Characteristics in a Mexican Population

**DOI:** 10.3390/ncrna12030019

**Published:** 2026-06-04

**Authors:** Mónica Alejandra Rosales-Reynoso, Anilú Margarita Saucedo-Sariñana, Clara Ibet Juárez-Vázquez, César de Jesús Tovar-Jácome, Rubria Alicia González-Sánchez, Karen Guadalupe Mestas-Villagran, Gustavo Andrés Torres-Sánchez, José Elías García-Ortíz, Efraín Salas-González, Martha Patricia Gallegos-Arreola

**Affiliations:** 1 División de Medicina Molecular, Centro de Investigación Biomédica de Occidente (CIBO), Centro Médico Nacional de Occidente (CMNO), Instituto Mexicano del Seguro Social (IMSS), Guadalajara 44340, Jalisco, Mexico; cesartovjacome@gmail.com (C.d.J.T.-J.); rubria18@gmail.com (R.A.G.-S.); mestas.karen@gmail.com (K.G.M.-V.); gandrestorres2803@gmail.com (G.A.T.-S.); 2 Departamento de Biología Molecular y Genómica, Centro Universitario de Ciencias de la Salud (CUCS), Universidad de Guadalajara (UdeG), Guadalajara 44340, Jalisco, Mexico; saucedo.anilu@gmail.com; 3 Departamento de Estructura y Función, Facultad de Medicina, Decanato de Medicina, Universidad Autónoma de Guadalajara (UAG), Zapopan 45129, Jalisco, Mexico; clara.juarez.vazquez@gmail.com; 4 División de Genética, Centro de Investigación Biomédica de Occidente (CIBO), Centro Médico Nacional de Occidente (CMNO), Instituto Mexicano del Seguro Social (IMSS), Guadalajara 44340, Jalisco, Mexico; jose.elias.garcia@gmail.com (J.E.G.-O.); marthapatriciagallegos08@gmail.com (M.P.G.-A.); 5 Servicio de Oncología Médica, Unidad Médica de Alta Especialidad, Hospital de Ginecología y Obstetricia, Centro Médico Nacional de Occidente (CMNO), Instituto Mexicano del Seguro Social (IMSS), Guadalajara 44340, Jalisco, Mexico; esgonco@hotmail.com

**Keywords:** lncRNA, breast cancer, Mexican population, *H19*, *MEG3*, *POLR2E*, *ANRIL*

## Abstract

Recent evidence has highlighted the crucial role of non-coding genetic elements in regulating gene expression and has been linked to a broad range of biological functions. Notably, dysregulation of long non-coding RNAs has been strongly associated with tumorigenesis and cancer progression. **Background/Objectives**: This study aimed to investigate the potential association between the *H19* rs3741219 T>C, *MEG3* rs7158663 G>A, *POLR2E* rs3787016 T>C, and *ANRIL* rs10757274 A>G variants and Breast Cancer (BC) susceptibility, as well as their relationship with clinicopathological characteristics in Mexican patients. **Methods**: DNA was obtained from peripheral blood samples of 505 women (254 patients and 251 control females). Genotyping was performed by polymerase chain reaction restriction fragment length polymorphism (PCR-RFLP) methodology. Associations were calculated using odds ratios (ORs), with *p*-values adjusted by the Bonferroni test (*p* < 0.012). In silico analyses were conducted to predict the functional impact of the variants associated. **Results**: Patients carrying the C/C genotypes in *H19* rs3741219 and *POLR2E* rs3787016 variants showed increased susceptibility to developing BC and with clinical and pathological characteristics (age at diagnosis, TNM stage, histologic type and molecular subtype) (*p* < 0.001). **Conclusions**: The results suggest that *H19* rs3741219 and *POLR2E* rs3787016 variants significantly influence BC risk.

## 1. Introduction

Breast cancer comprises a biological and molecular group of heterogeneous diseases that originate in breast tissue due to dysregulation of pathways controlling cell proliferation and apoptosis. These alterations promote a complex multistep carcinogenic process leading to malignant transformation [1,2]. Globally, BC continues to represent a major public health challenge. Data from GLOBOCAN 2022 indicate that it accounted for 2,296,840 newly diagnosed cases and 666,103 deaths, making BC the fifth leading cause of cancer-related mortality and the most diagnosed malignancy in women [3]. In Mexico, 29,929 new cases and 7931 deaths were reported in the same year, making BC the most prevalent malignancy and the primary cause of cancer-related mortality among Mexican women [4].

The development and clinical behavior of BC results from a complex interplay of genetic and environmental factors [5]. Beyond well-established breast cancer susceptibility genes such as *BRCA1*, *BRCA2*, *PALB2*, *CHEK2*, and *TP53*, accumulating evidence indicates that non-coding regulatory elements, particularly long non-coding RNAs (lncRNAs), also contribute to breast cancer susceptibility and tumor heterogeneity. Recent large-scale genomic studies have highlighted that many breast cancer risk variants are enriched within non-coding regions, where they may modulate gene expression, chromatin dynamics, and oncogenic pathways [6,7,8].

LncRNAs are transcripts longer than 200 nt that are transcribed like mRNAs but are not processed into protein [9]. Recently, they have gained attention due to their emerging role in gene regulation and tumorigenesis. LncRNAs have been implicated in key cellular processes such as transcriptional and translational regulation, chromatin modification, nuclear organization, and cytoplasmic signaling [9,10,11]. Through these mechanisms, lncRNAs have been implicated in breast carcinogenesis, disease progression, and therapeutic response, highlighting their potential utility [6]. Nevertheless, the precise molecular mechanisms by which lncRNAs contribute to BC development remain incompletely understood [12,13].

Among the most studied lncRNAs is *H19*, a 2.3 kb oncofetal transcript located on chromosome 11p15.5 that is maternally expressed and paternally imprinted. This gene is approximately 35 kb in size and contains five exons. *H19* is overexpressed and implicated in malignant conditions, including BC, lung cancer, glioma, and cholangiocarcinoma [14,15,16,17,18]. *H19* expression varies according to clinical and histological subtype, suggesting a role that is influenced by tumor context [16]. It contributes to BC tumorigenesis and progression through diverse mechanisms, including encoding microRNA-675, regulating gene expression as a competing endogenous RNA (ceRNA) sequestering other microRNAs, and interacting with oncogenic regulators such as MYC, thereby affecting cell proliferation and survival [19]. The rs3741219 A>G variant in the *H19* gene may create a binding site for hsa-miR-1539 and alter the stability and secondary structure of the lncRNA, potentially affecting miRNA-lncRNA interactions [16]. Notably, the rs3741219 A>G variant demonstrates cancer type-specific associations [17,20,21,22,23]. In hepatocellular and ovarian cancer, it has been linked to an increased risk and poorer prognosis, whereas in glioma, the same variant appears to exert a protective effect [21,22]. However, in other cancers such as breast cancer [24], gastric [25], colorectal [24], lung [26], cervical [20], and bladder [24], results remain inconclusive.

Another relevant lncRNA is *ANRIL* (Antisense Non-Coding RNA in the *INK4* Locus), also known as *CDKN2B*-*AS*, located in the *CDKN2A*/*B* gene cluster on chromosome 9p21.3. This gene comprises 19–21 exons and spans approximately 196 kb. *ANRIL* regulates gene expression through chromatin modification, repressing the expression of neighboring tumor suppressor genes *CDKN2A* and *CDKN2B*, which increases tumorigenesis [27]. Recent evidence has shown an elevated *ANRIL* expression in BC patients compared to healthy controls. *ANRIL* demonstrates variable expression according to BC subtype and indicates a potential role in disease progression and its utility as a biomarker for clinicopathological staging [10,28,29]. Variants of *ANRIL*, such as rs10757274 A>G, have been analyzed in cardiovascular disease and several types of cancer, including BC [27,30,31,32]. Although no association was observed in BC [31], some haplotypes, including rs10757274, have been associated with increased susceptibility to BC risk [30].

The polymerase II polypeptide E *(POLR2E)* gene is located on chromosome 19p13.3, contains nine exons and encodes the fifth-largest subunit of RNA polymerase II [33]. *POLR2E* has been associated with increased susceptibility in several cancers, including prostate, esophageal, papillary thyroid, and BC [34]. The rs3787016 C>T variant is an intronic SNV located within intron 4 of *POLR2E* and has been associated with increased susceptibility to various malignancies, including thyroid [35], gastric [36], prostate [37], and BC [34,38].

On the other hand, *MEG3* (Maternally Expressed Gene 3), located on chromosome 14q32.3 within the *DLK1*-*MEG3* locus and spanning approximately 1.6 kb, is regarded as a tumor suppressor gene [39,40]. Its proposed effect is exerted primarily through the activation of the p53 pathway, often via hypermethylation [39,41]. Reduced expression of *MEG3* has been documented in various cancers, including gliomas, hepatocarcinoma, colorectal, cervical, and BC [39]. Bioinformatic analysis revealed that the rs7158663 G>A variant alters the secondary structure of *MEG3*, potentially disrupting its regulatory interactions. Thus, rs7158663 may represent a functional regulatory SNP capable of modulating *MEG3* expression and contributing to cancer susceptibility [41]. The rs7158663 G>A variant has been associated with altered *MEG3* expression and increased susceptibility to several cancers, including BC [39,42,43].

LncRNAs have been extensively studied because they do not overlap with protein-coding regions. However, SNVs located in lncRNAs that overlap protein-coding genes may simultaneously impact both lncRNA regulatory activity and protein function. Moreover, recent studies suggest that these SNVs can disrupt RNA folding and alter miRNA or protein binding, thereby affecting cancer-related pathways [34,35]. Although the clinical application of these variants remains to be fully established, identifying lncRNA-related variants associated with BC susceptibility may improve our understanding of genetic risk factors and contribute to future personalized screening and prevention strategies [6].

In this context, this study aimed to investigate, for the first time, the association of *H19* rs3741219 T>C, *ANRIL* rs10757274 A>G, *POLR2E* rs3787016 C>T, and *MEG3* rs7158663 G>A variants with breast cancer susceptibility in Mexican patients. These variants have shown potential functional relevance and associations with cancer susceptibility in different populations; however, their contribution to BC risk remains inconsistent across ethnic groups and has been poorly explored in Latin American populations. Additionally, this study evaluated their potential relationship with clinicopathological features in Mexican patients.

## 2. Results

### 2.1. Characteristics of the Subjects Included in the Study

Table 1 shows a comparative analysis of demographic and clinicopathological characteristics of BC patients and the control group. The mean age observed was 47.15 and 46.60 years for the BC and control group, respectively. In the age-stratified analysis, a statistically significant difference was observed between the groups (*p* < 0.001). Alcohol consumption was not associated (*p* = 0.831), while tobacco consumption showed an association with breast cancer (*p* < 0.001). The BMI observed in the BC group was overweight (27.29). According to clinicopathological characteristics observed in the BC group, 65.4% were in advanced stages III-IV, 94.9% had unilateral tumors, 87.4% had ductal type cancer, 56.3% were luminal type A, and 30.7% were lymph node-positive.

### 2.2. Genotypic and Allelic Frequencies Analysis of the Variants Studied

Table 2 shows the comparative analysis of the genotypic and allelic frequencies for the variants *MEG3* rs7158663, *POLR2E* rs3787016, *ANRIL* rs10757274 and *H19* rs37741219 in BC and the control group. In the control group, the four analyzed SNVs were observed in Hardy–Weinberg equilibrium. Statistical significance was observed for the *H19* rs3741219 (T>C) and *POLR2E* rs3787016 (T>C) variants in the BC patients. For the *MEG3* rs7158663 (G>A) and *ANRIL* rs10757274 (A>G) variants, no statistically significant differences were observed. For the *H19* rs3741219 (T>C) variant, we observed that the patients carriers of C/C genotype showed an increased susceptibility for developing breast cancer (OR = 2.41; 95% CI = 1.42–4.09, *p* = 0.001); this association was also evident under the dominant model of inheritance (T/C + C/C vs. T/T) (OR = 1.84; 95% CI = 1.26–2.69, *p* = 0.001). Allelic frequencies were also significantly different, demonstrating that carriers of the C allele have increased susceptibility for developing BC (OR = 1.55; 95% CI = 1.21–2.00, *p* = 0.001). Regarding the *POLR2E* rs3787016 (T>C) variant, patients carriers of C/C genotype shown an increased susceptibility for developing BC (OR = 2.08; 95% CI = 1.21–3.58, *p* = 0.019); this association was also evident under the dominant model of inheritance (T/C + C/C vs. T/T) (OR = 1.54; 95% CI = 1.06–2.22, *p* = 0.025). Also, allelic frequencies were significantly different, demonstrating that carriers of the C allele have increased susceptibility for developing breast cancer (OR = 1.42; 95% CI = 1.10–1.83, *p* = 0.007).

### 2.3. Association of H19, POLR2E, ANRIL and MEG3 Genotypes with Demographic and Clinicopathological Characteristics

The stratification analysis by age, smoking, alcohol consumption, TNM stage, histologic type, and histologic–molecular subtype for the *H19* rs3741219, *POLR2E* rs3787016, *ANRIL* rs10757274, and *MEG3* rs7158663 variants is shown in Table 3, Table 4, Table 5 and Table 6. Concerning the *H19* rs3741219 variant, we observed that patients who were carriers of the C/C genotype and under 50 years old showed an increased susceptibility to developing BC (OR 2.87; 95% CI = 1.44–5.70, *p* = 0.003) (Table 3).

In the stratification analysis by TNM stage, histologic type, and histologic–molecular subtype, in patients with the *H19 rs374119* variant, we found that patients with an early TNM stage (I + II) and carriers of the C/C genotype showed an increased susceptibility (OR 3.36; 95% CI 1.66–6.79, *p* = 0.001). Regarding the ductal histologic type, we observed that patient carriers of T/C and C/C genotypes show an increased susceptibility to develop breast cancer (OR = 1.95; 95% CI = 1.28–2.98, *p* = 0.002 and OR = 2.88; 95% CI = 1.66–4.99, *p* = 0.001), respectively. For the histological–molecular subtypes, an increased susceptibility was observed in patients with luminal A and B subtypes and carrying C/C genotypes (OR = 2.26; 95% CI = 1.22–4.18, *p* = 0.013 and OR = 3.18; 95% CI = 1.32–7.64, *p* = 0.014), respectively (Table 3).

Regarding the *POLR2E* rs3787016 variant, we observed that patient carriers of the C/C genotype and over 50 years old showed an increased susceptibility to develop BC (OR 2.66; 95% CI = 1.28–5.54, *p* = 0.013) (Table 4). In the analysis by TNM stage, histologic type, and histologic–molecular subtype, we found that the patients with early TNM stage and carriers of C/C genotype showed an increased susceptibility (OR 3.15; 95% CI = 1.65–5.99, *p* = 0.001). In the ductal histologic type, carriers of C/C genotype show an increased susceptibility to developing breast cancer (OR = 2.61; 95% CI = 1.61–4.21, *p* = 300.001), while for the histologic–molecular subtypes, statistical significance was observed for luminal A subtype in the patients carrying the C/C genotype (OR = 2.00; 95% CI = 1.07–3.71, *p* = 0.039) (Table 4).

Concerning the *MEG3* rs7158663 and *ANRIL* rs10757274 variants, no statistically significant differences were observed when comparing the genotypes with the clinicopathological characteristics (Table 5 and Table 6).

### 2.4. Multivariable Logistic Regression Analysis with Variables Associated

The results of the multiple logistic regression analysis are presented in Table 7. Statistical significance was observed for age (>50 years) and tobacco consumption in the presence of two variants associated with *H19* rs3741219 and *POLR2E* rs3787016 (OR = 1.78; 95% CI = 1.20–2.63; *p* = 0.004 and OR = 1.52; 95% CI = 1.04–2.22; *p* = 0.030), respectively, suggesting that those variables increase the risk and susceptibility to developing BC.

### 2.5. In Silico Analysis of Variants H19 rs3741219 and POLR2E rs3787016

According to the CADD analysis, the *H19* rs3741219 variant received a PHRED score of 9.365 and a raw score of 0.9108, suggesting that the variant may possibly have a deleterious effect. The GERP conservation score of 5.24 further supports its evolutionary conservation, suggesting functional relevance.

**Figure 1:**
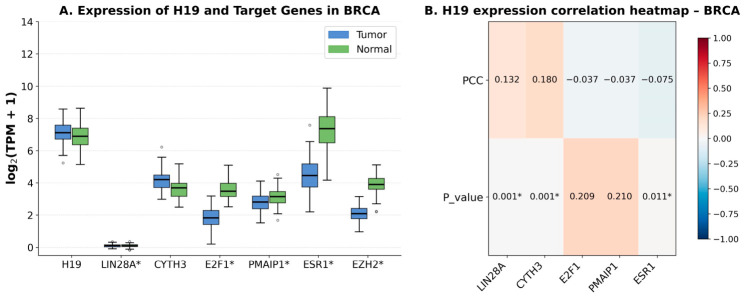
Expression and correlation analysis of *H19* and predicted target genes in BRCA. (**A**). Boxplots showing the expression levels (log_2_[TPM+1]) of *H19* and its predicted target genes in BRCA tumor and normal tissues. Genes marked with an asterisk (*) exhibit statistically significant differential expression. Circles (○) represent outliers (**B**). Heatmap illustrates the Pearson correlation coefficients (PCCs) and corresponding *p*-values between *H19* and selected target genes in BRCA. * *p* < 0.05. Color intensity ranges from negative/weak correlation to red positive/strong correlation.

As illustrated in Table 8, *H19* exhibits an intricate interaction with various genes associated with BC, including *ESR1*, *EZH2*, *PMAIP1*, *HOTAIR*, *91H*, *E2F1*, *CYTH3*, *LIN28A*, *CBL*, and *DNMT*. Figure 1 presents the expression profiles of *H19*, along with the predicted targets of *H19*, and a heatmap illustrating the correlation coefficients for each H19–target pair. Among the genes evaluated, five targets were identified. As demonstrated in Figure 1A, *LIN28A*, *E2F1*, *PMAIP1*, *ESR1*, and *EZH2* exhibited statistically significant differential expressions. As demonstrated by the correlation heatmap, there were statistically significant associations for *LIN28A*, *CYTH3*, and *ESR1*. *LIN28A* and *CYTH3* displayed weak positive correlations with *H19*, whereas *ESR1* exhibited a slight negative correlation (see Figure 1B).

Regarding the *POLR2E* rs3787016 variant, CADD analysis showed that this variant has a PHRED score of 0.289 and a raw score of –0.331. The GERP conservation score was 1.73, and the variant was annotated within a transcription factor binding site (TFBS). Figure S1 shows the identification and characterization of candidate transcription factors that can bind to the *POLR2E* variant site.

Figure S1A illustrates the genomic mapping where the variant overlaps with the binding coordinates of AGO2, RBFOX2, and POLR2A. Figure S1B demonstrates the signal intensity values (relative binding strength), with POLR2A exhibiting the most substantial enrichment (10^9^), followed by RBFOX2 (8.6 × 10^2^) and AGO2 (2.1 × 10^2^). Figure S1C describes the tissue or cell line origin of each transcription factor: POLR2A in spleen tissue, RBFOX2 in the K562 cell line (blood), and AGO2 in the HepG2 cell line (liver).

Immunohistochemistry data retrieved from The Human Protein Atlas (an independent external cohort not characterized for the SNPs of interest in our study) were reviewed to assess the protein expression of the transcription factors identified through RegulomeDB. RBFOX2 was detected in 67% (8/12) of BC samples, predominantly in ductal carcinoma (89%) with low staining intensity. AGO2 was detected in all cases (12/12), with high intensity in 25% (3/12, all lobular), moderate in 67% (8/12; 88% lobular, 12% ductal), and low in 8% (1/12, lobular). POLR2A immunohistochemistry data were not available (Table S1). These results provide orthogonal evidence of biological plausibility at the protein level rather than direct evidence of a genotype–phenotype association in our population.

### 2.6. In Silico Analysis of H19 and POLR2E Gene Expression

**Figure 2:**
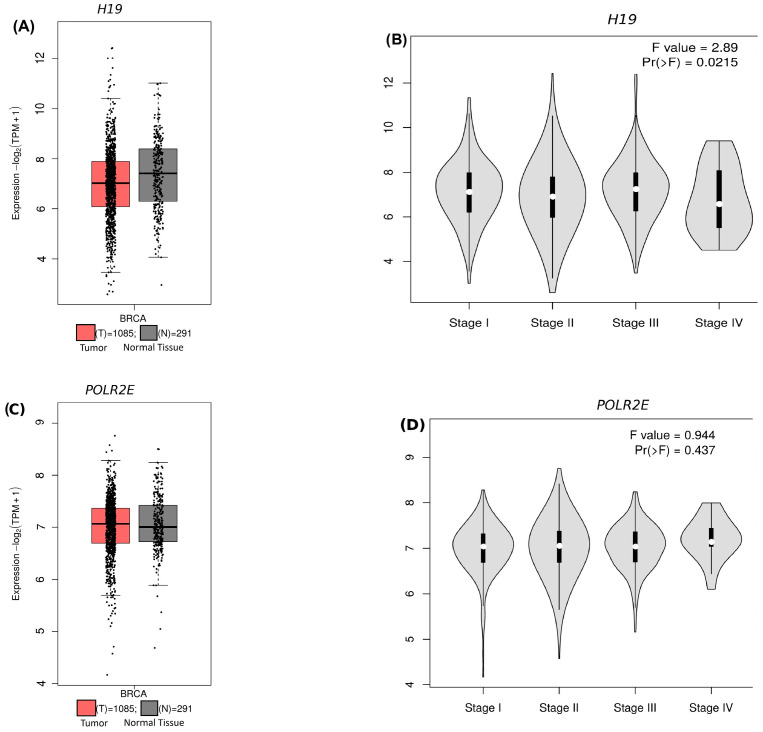
Expression analysis of *H19* and *POLR2E* in BC and across clinical stages (GEPIA2). (**A**,**C**) Boxplots showing expression levels (log_2_[TPM+1]) of *H19* and *POLR2E*, respectively, comparing breast cancer tumor samples with normal breast tissue. (**B**,**D**) Violin plots illustrating stage-specific expression patterns for *H19* and *POLR2E* across BC stages I, II, III, IV.

Gene expression profiling was performed, integrating RNA-Seq expression data from repositories, to evaluate the behavior of these genes in breast cancer tissue from patients. Figure 2 illustrates the gene expression profiling of *H19* and *POLR2E* among tissue types and tumoral stages. Figure 2A presents the differential expression analysis, where *H19* exhibited no statistically significant differences between tumor and normal breast tissue, and Figure 2B shows that stage-specific expression analysis of *H19* demonstrated significant variability across clinical stages (F = 2.89; *p* = 0.02), with underexpression observed in stage IV. Finally, Figure 2C shows differential expression analysis of *POLR2E*, which showed no statistically significant differential expression between tumor and normal breast tissue. Figure 2D indicates that stage-specific expression analysis of *POLR2E* detected no considerable variation in expression levels (F = 0.944; *p* = 0.43).

### 2.7. In Silico Analysis of Survival of H19 and POLR2E

Kaplan–Meier survival analyses were performed using RNA-seq data from online repositories to evaluate the association of *H19* and *POLR2E* expression with overall survival in BC patients.

**Figure 3:**
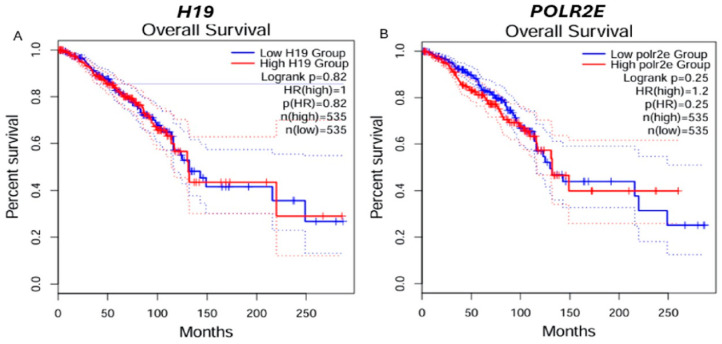
Overall survival according to *H19* and *POLR2E* expression levels in breast cancer (GEPIA2). (**A**,**B**). Kaplan–Meier curves comparing overall survival between patients with low (**blue**) and high (**red**) expression of *H19* (**left**) and *POLR2E* (**right**). Each panel displays the number of patients per group (n), the log-rank *p*-value, and the hazard ratio (HR) with its corresponding *p*-value for the high-expression group.

Figure 3 presents the Kaplan–Meier survival analysis performed based on *H19* and *POLR2E* expression. Figure 3A shows that in overall survival in breast cancer for *H19*, this gene has no statistically significant differences in overall survival between patients with high and low expression of this gene (log rank *p* = 0.82; HR = 1.00; p(HR) = 0.82). Finally, Figure 3B indicates overall survival for *POLR2E*; this survival curve revealed no statistically significant differences between high and low expression groups of this gene (log rank *p* = 0.25; HR = 1.20; p(HR) = 0.25).

## 3. Discussion

In the present study, the association between four genetic variants located in long non-coding RNAs (*H19* rs3741219, *MEG3* rs7158663, *POLR2E* rs3787016, and *ANRIL* rs10757274) and BC susceptibility was evaluated in Mexican mestizo women, integrating the clinicopathological characterization of the cohort with in silico functional analyses. Two main findings can be highlighted; on one hand, we observed that the C/C genotypes of *H19* rs3741219 and *POLR2E* rs3787016 were significantly associated with increased BC susceptibility, while specific clinicopathological associations were also found, with early-onset disease and luminal B subtype for *H19* and late-onset disease and luminal A subtype for *POLR2E*. On the other hand, the in silico analyses showed that both variants are located within regions that have regulatory relevance, although with different levels of predicted intrinsic impact, so that biological plausibility for their phenotypic associations is supported. For the *MEG3* rs7158663 and *ANRIL* rs10757274 variants, no significant association with BC susceptibility was observed in our population. To our knowledge, this is the first work in which these four lncRNA variants are characterized in an admixed Latin American population, as population-specific data in the literature is mostly derived from Asian and European cohorts, and a recognized gap in the genetic architecture of BC susceptibility in Latin American women is addressed.

### 3.1. Demographic and Clinicopathological Context

In our cohort, BC incidence was statistically more frequent in patients aged 50 years and above, which is consistent with the international and national guidelines (ESMO, NICE, NCCN, NOM), in which age is considered a major risk factor for BC [44,45,46]. This pattern can be explained by the cumulative accrual of genetic damage in breast tissue throughout life, the prolonged hormonal exposure to estrogen and progesterone during reproductive years, and the age-related decline in immune surveillance [47,48]. Additionally, a statistically significant association with cigarette smoking was observed, which is in line with the IMSS guidelines and the previous evidence on the carcinogenic effects of the chemical compounds present in tobacco smoke, including polycyclic hydrocarbons, nitrosamines, and aromatic amines [49,50]. Thus, the demographic and behavioral patterns observed in our population provide the clinical context in which the genetic associations described below should be interpreted.

### 3.2. H19 rs3741219 Variant: Results in Context

Our results show that patients carrying the T/C or C/C genotypes of *H19* rs3741219 exhibited an increased susceptibility to developing BC, particularly with early-onset disease and luminal A and luminal B subtypes. These findings contrast with reports by Xia et al. (2016) [51] and Abdollahzadeh et al. (2019) [52], who found no association in Chinese and Iranian populations, and with meta-analyses by Li et al. (2022) [53], Yuan et al. (2022) [54], and Yang et al. (2023) [22] reporting no overall association with cancer risk. The discrepancies likely reflect genetic heterogeneity across populations and differences in environmental exposures.

The functional plausibility of *H19* rs3741219 is supported by its location within exon 1, a region essential for the regulatory activity of the transcript, together with a high GERP conservation score (5.24) and a modestly elevated CADD PHRED score, indicating that this position is under evolutionary constraint and may harbor regulatory relevance. Among the *H19* targets retrieved from LncTarD, only *LIN28A*, *CYTH3*, and *ESR1* showed significant expression correlations in TCGA-BRCA data, with *ESR1* displaying an inverse association. This pattern is consistent with our observation of increased susceptibility in luminal A and luminal B carriers of the C/C genotype, as *H19* has been described as an estrogen-responsive transcript whose expression is induced by ERα in luminal progenitor cells [54]. Within this framework, the rs3741219 variant may modulate *H19*-mediated regulation of hormone-responsive programs, providing a biologically coherent, though not definitive, explanation for the subtype-specific signal observed in our cohort. Other reported functions of *H19*, including its roles in EMT, stemness, and endocrine resistance, were not assessed in the present study and therefore cannot be linked directly to our findings.

### 3.3. POLR2E rs3787016 Variant: Results in Context

Patients carrying the C/C genotype of *POLR2E* rs3787016 showed increased BC susceptibility, with the strongest signal in women > 50 years and in luminal A tumors. These results are consistent with those reported by Xu et al. (2017) [38] and Chen et al. [34], although the subtype-specific association differs from Xu et al., who reported T/T as the susceptibility genotype for ER/PR-positive tumors in a different population. Multivariate analysis confirmed age as an independent susceptibility factor in our cohort, in line with established age-related mechanisms in BC pathogenesis [45,46].

In contrast to *H19* rs3741219, in silico prediction scores for *POLR2E* rs3787016 did not indicate a strong intrinsic functional impact (PHRED 0.289; GERP 1.73), suggesting that if this variant is causal, its effect is likely subtle and context-dependent. The most parsimonious interpretation compatible with our data is that rs3787016 overlaps a transcription factor binding region with documented occupancy by POLR2A, RBFOX2, and AGO2, proteins involved in transcriptional and post-transcriptional regulation, and RBFOX2 and AGO2 were detectable in breast tissue by immunohistochemistry in The Human Protein Atlas, supporting the biological plausibility of a regulatory effect at this locus. An alternative possibility, which our study cannot exclude, is that rs3787016 is not the causal variant but rather a marker in linkage disequilibrium with a yet-unidentified functional variant. Taken together, the evidence supports a context-dependent role for rs3787016 in BC susceptibility, most apparent in women over 50 years of age and in luminal A tumors, rather than a strong, generalized functional effect.

### 3.4. MEG3 rs7158663 and ANRIL rs10757274: Absence of Association

For the *MEG3* rs7158663 and *ANRIL* rs10757274 variants, no significant associations with BC susceptibility were observed in our population. Regarding *ANRIL* rs10757274, this negative result is consistent with what has been previously reported in BC, where no clear association has been found despite the well-documented role of *ANRIL* in the epigenetic regulation of the *CDKN2A/CDKN2B* tumor-suppressor locus [27,29,30,31]. For *MEG3* rs7158663, however, prior evidence has been mixed, while some studies have suggested an association with reduced *MEG3* expression and increased cancer susceptibility [39,41,43]. The absence of association in our cohort may be explained by insufficient statistical power to detect modest effects, ancestry-specific allele frequencies, or a genuine lack of association in Mexican mestizo women, so that replication in larger Latin American cohorts is needed to clarify the role of these variants in our population.

### 3.5. Clinical Relevance and Translational Considerations

Although our findings identify *H19* rs3741219 and *POLR2E* rs3787016 as susceptibility variants in Mexican mestizo women, several considerations should be taken into account before their clinical translation can be considered. The effect sizes observed in our analyses (OR ~1.5–2.4) are characteristic of low-to-moderate penetrance variants, so that limited discriminatory power would be provided if used as standalone biomarkers. A more realistic clinical incorporation would occur through their integration into population-specific polygenic risk scores (PRS), in combination with high- and moderate-penetrance gene panels (*BRCA1*, *BRCA2*, *PALB2*, *ATM*, *CHEK2*, *TP53*) and the established non-genetic risk factors that are routinely used in the clinical setting, such as age, BMI, reproductive history, and family history of cancer.

This integration is particularly relevant in Latin American populations, in which the PRS (Polygenic Risk Score) models that are currently available, mostly developed in European-ancestry cohorts, have shown a substantially poorer performance when applied to admixed populations. Recent evaluations of breast cancer PRS panels in Hispanic/Latinx women have shown lower discriminatory accuracy (AUC ~0.61–0.63) when compared to European-ancestry cohorts, while the performance was found to vary according to the degree of Indigenous American ancestry of the participants [55,56]. Additionally, multi-ancestry PRS (MA-PRS) approaches have shown improved performance in Hispanic women, particularly for the prediction of early-onset and triple-negative BC, although these approaches still require further validation before being incorporated into routine clinical use [57]. Therefore, the identification of ancestry-relevant variants like those characterized in the present study represents one step towards closing this gap.

Several barriers should still be overcome before any clinical implementation can be considered, including the independent validation of these results in additional Mexican and broader Latin American cohorts; the functional characterization of the regulatory consequences of each variant through experimental approaches such as allele-specific expression assays, reporter constructs, and CRISPR-based perturbations; and the cost-effectiveness analyses needed for any population-level screening strategy. Additionally, the development of clinical-grade genotyping platforms that incorporate ancestry-informative markers represents another step required for a meaningful implementation.

### 3.6. Limitations and Future Directions

Several limitations should be considered when interpreting these findings. The associations identified correspond to a case–control comparison and reflect susceptibility to breast cancer development; they do not imply causality and should not be interpreted as prognostic markers. Consistent with this distinction, *H19* and *POLR2E* expression did not differ significantly between tumor and normal tissue in the TCGA-BRCA cohort, and neither was associated with overall survival—an expected result if the susceptibility signal operates at the level of disease initiation rather than tumor progression. Moreover, bulk transcriptomic analyses average expression across heterogeneous cell populations and may dilute cell type- or subtype-specific regulatory effects that are particularly relevant for lncRNAs, whose expression is often spatially and temporally restricted. The absence of patient-matched expression data within our own cohort, together with the lack of survival follow-up, also limits the ability to integrate genetic and transcriptional findings in the same individuals; this should be regarded as a constraint of the present study. Functional validation in larger, ethnically diverse cohorts and in subtype-specific cellular models will be needed to clarify the mechanistic contribution of these variants to BC susceptibility.

## 4. Materials and Methods

### 4.1. Subjects

A total of 505 women were recruited, including 254 patients with a clinical and histological diagnosis of BC, based on the criteria of the UMAE Hospital de Gineco-Obstetricia of the Instituto Mexicano del Seguro Social (IMSS) in Guadalajara, Mexico. BC was stratified according to the tumor–node–metastasis (TNM) classification. The study was approved by the National Committee for Scientific Research of the Mexican Institute of Social Security (IMSS) (R-2020-785-130) with approval date August 2020 and conducted following national and international ethical standards. The patient group comprised women aged 18 years or older with any stage of BC, regardless of treatment status or therapeutic stage. The control group included 251 unrelated healthy women donors aged 18 years or older from the general Mexican population, matched for age with the patient group. Patients and healthy women were enrolled in a non-probabilistic consecutive manner during consultation at this institution between 2020 and 2023. All the participants provided written informed consent for participation in this study. In addition, this study was performed in accordance with the Declaration of Helsinki and its latest amendments. A standard epidemiological questionnaire allowed us to collect personal data, including age, drinking and smoking status, and familial history for all participants. Women with a history of previous cancer were excluded from the control group, while those who had undergone chemotherapy or radiotherapy were excluded from the patient group. Information about the clinical and pathological characteristics of patients was obtained from hospital records.

### 4.2. Genotyping

Genomic DNA from peripheral blood lymphocytes was isolated by the salting-out method [58]. The variants *H19* rs3741219 (T>C), *POLR2E* rs3787016 (T>C), *MEG3* rs7158663 (G>A), and *ANRIL* rs10757274 (A>G) were genotyped using the polymerase chain reaction–restriction fragment length polymorphism (PCR-RFLP) methodology with the following primer pairs: for the *H19* rs3741219 variant, the forward primer F: 5′-CCC CCT GCG GCG GAC GGT TGA-3′ and reverse primer R: 5′-GGC GTA ATG GAA TGC TTG AA-3′ [59]; for the *POLR2E* rs3787016 variant F: 5′-CAT CAA CAT CAC GCA GCA CG-3′ and reverse primer R: 5′-CCC TGT CCT CCA AGC ACT CAT-3 [38]; for *ANRIL* rs10757274 F: 5′-GTT TCT GCA CAT GGT GAT GG-3′ and reverse primer 5′-CTGCCTCACTCTCCAGTTCC-3′ [60]; and for the *MEG3* rs7158663 variant, the forward primer F: 5′-GGT TCT TTA GTT CTG CGA TGC T-3′ and the reverse primer 5′-TTG GGA GTC ACA AGA GGA GG-3′ were used [61].

PCR for the *MEG3* rs7158663, *POLR2E* rs3787016, *ANRIL* rs10757274 and *H19* rs37741219 variants was performed for 35 cycles in a 10 μL volume containing 100 ng DNA, 10X buffer (500 mM KCl, 100 mM Tris- HCl, and 0.1% Triton TMX-100) (Invitrogen, Carlsbad, CA, USA), 2.0 mM MgCl_2_, 200 μM dNTPs, 5 pM of each primer, and 2 U Taq DNA Polymerase. Denaturation was carried out at 95 °C, annealing temperature was set at 60 °C for rs7158663, 59 °C for rs3787016, 60 °C for rs10757274, and 62 °C for rs3741219, and elongation at 72 °C for 2 min each. All PCR reactions were performed in a thermocycler T-100 Biorad (Biorad, California, USA). Five microliters of the PCR product were digested with 4U of *BtsC*I, *Nla*III, *BsmA*I, and *Hha*I, restriction enzymes (New England Bio-Labs Inc; Beverly, MA, USA), respectively. The digested products were separated into 8% polyacrylamide gels. For *MEG3* rs7158663, the fragment sizes observed by electrophoresis corresponded to 233 and 68 bp for the polymorphic allele (A) and 301 bp for the wild-type allele (G). For the *POLR2E* rs3787016 variant, the fragment sizes observed by electrophoresis corresponded to 127 and 20 bp for the polymorphic allele (C) and 147 bp for the wild-type allele (T); for the *ANRIL* rs10757274 variant, the fragment sizes observed were 250 bp for the polymorphic allele (G) and 172 and 78 bp for the wild-type allele (A); and finally, for the *H19* rs3741219 variant, the fragment sizes observed were 301, 92 and 41 bp for the polymorphic allele (C) and 342 and 92 bp for the wild-type allele (T).

Quality control for these assays was assessed in 20% of randomly selected samples that were re-genotyped by an independent technician. The concordance among genotype assays was 100%.

### 4.3. Statistical Analysis

Allele and genotype frequencies were estimated by direct counting in both groups. The chi-square test was used to assess the Hardy–Weinberg equilibrium (HWE). The chi-square test was used to examine the differences in allele and genotype distributions and clinical characteristics between patients and controls. To measure the association of alleles or genotypes with clinicopathological characteristics, we performed a stratified analysis including age, smoking and alcohol status, TNM stage, and histologic–molecular subtype, and the odds ratio (OR) with corresponding 95% confidence intervals (CIs) using SPSS v17.0 software package (SPSS, Inc., Chicago, IL, USA). Multivariable logistic regression analysis was performed to evaluate confounding variables. *p*-values were adjusted using the Bonferroni method. Statistical significance was defined as *p* < 0.012 after adjustment.

### 4.4. In Silico Analysis of Genetic Variants

The estimated functional impact of the analyzed variants was assessed using complementary in silico tools, applied exclusively to the two variants that showed statistically significant association with breast cancer susceptibility in our experimental analysis. As a first step, CADD v1.7 (https://cadd.gs.washington.edu, accessed on 15 March 2026) [62,63] was used as the principal marker of deleteriousness, since it integrates more than sixty annotation features into a single PHRED-scaled score, including evolutionary conservation metrics, regulatory elements, transcription factor binding sites, chromatin marks, and splicing predictions; thus, it remains applicable to non-coding variants whose operational characteristics differ from those of coding variants. Based on the regulatory annotations retrieved from CADD, complementary tools were applied selectively according to the functional context of each variant. For variants overlapping a transcription factor binding site, RegulomeDB (https://regulomedb.org, accessed on 15 March 2026) [64] was queried to identify transcription factors with experimental ChIP-seq evidence at the locus, while the Human Protein Atlas (https://proteinatlas.org, accessed on 16 March 2026) [65] was consulted to confirm expression of these regulators in breast tissue. For variants located within a long non-coding RNA gene with documented target activity, curated lncRNA–target interactions in breast cancer were retrieved from LncTarD 2.0 (https://lnctard.bio-database.com, accessed on 17 March 2026) [66]. Custom figures derived from these analyses, including locus maps, correlation heatmaps, and grouped boxplots, were generated in R version 4.3.2 using the ggplot2 and ggpubr packages and were exported in TIFF format at 300 dpi.

### 4.5. In Silico Gene Expression and Survival Analysis

To support the in silico analysis with publicly available expression data, we utilized the GEPIA2 platform (http://gepia2.cancer-pku.cn accessed on 15 March 2026) [67], which integrates RNA-seq data from TCGA and GTEx. We used the TCGA-BRCA cohort (1089 primary breast tumor samples and 113 matched normal breast tissue samples; RNA-seq, log_2_[TPM+1]-transformed) as the original reference dataset to compare expression levels between tumor and normal tissue and across clinical stages. Boxplots, violin plots, and Kaplan–Meier curves indicated in the figures were created on the GEPIA2 platform. All GEPIA2 analyses were conducted in March 2026.

## 5. Conclusions

In conclusion, our results indicate that the C/C genotypes of *H19* rs3741219 and *POLR2E* rs3787016 are associated with an increased susceptibility to develop breast cancer in Mexican mestizo women, and distinct clinicopathological patterns were observed for each variant, such that *H19* was associated with early-onset disease and luminal A and luminal B subtypes, while *POLR2E* was associated with late-onset disease and luminal A subtype. No significant association was observed for *MEG3* rs7158663 and *ANRIL* rs10757274 in our population. The in silico analyses support the biological plausibility of these associations, with stronger evidence for *H19*, given its higher evolutionary conservation and its documented regulatory interactions with luminal-related targets, while the contribution of *POLR2E* rs3787016 appears to be more context-dependent and likely mediated through transcription factors binding at this locus. These findings provide the first characterization of these variants in an admixed Latin American population and represent candidate markers for future incorporation into ancestry-informed risk stratification frameworks. Functional validation in larger cohorts and in subtype-specific cellular models will be necessary to confirm the mechanistic role of these variants and to evaluate their potential clinical utility.

## Figures and Tables

**Table 1:** Clinicopathological data of BC patients.

Characteristics	Breast Cancer Group N = 254 (100%)	Control Group N = 251 (100%)	*p* Value
**Mean Age** (years SD)	47.15 (±8.27)	46.60 (±9.42)	0.48
Age			
<50	130 (51.2)	17 (68.1)	**0.001**
>50	124 (48.8)	80 (31.9)	
**Consumption of Tobacco**			
Yes	66 (26.0)	35 (13.9)	**0.001**
No	188 (74.0)	216 (86.1)	
**Consumption of Alcohol**			
Yes	34 (13.4)	32 (12.7)	0.831
No	220 (86.6)	219 (87.3)	
**Body Mass Index (BMI)**			
mean	29.27 (±5.63)		
**Breastfeeding**			
No	60 (23.6)		
Yes	194 (76.4)		
<6 months	37 (14.6)		
>6 months	157 (61.8)		
**Hysterectomy**			
Yes	57 (22.4)		
No	197 (77.6)		
**TNM Stage**			
I + II	88 (34.6)		
III + IV	166 (65.4)		
**Tumor location**			
**Bilateral**	13 (5.1)		
**Unilateral**	241 (94.9)		
Left	131 (51.6)		
Right	110 (43.3)		
**Histology** (adenocarcinoma)			
Ductal	222 (87.4)		
Lobular	29 (11.4)		
Mixed	(1.2)		
**Molecular subtype**			
Luminal A	143 (56.3)		
Luminal B	58 (22.8)		
Her2	33 (13.0)		
Triple-negative	20 (7.9)		
**Metastatic lymph node status (N+)**			
Positive	78 (30.7)		
Negative	176 (69.3)		
**Metastasis(M+)**			
Yes	88 (34.6)		
No	166 (65.4)		

Bold text highlights statistically significant findings. SD: standard deviation. *p* values were adjusted by Bonferroni test (0.012).

**Table 2:** Distribution of genotypes and allelic frequencies of *H19* rs3741219, *POLR2E* rs3787016, *MEG3* rs7158663, and *ANRIL* rs10757274 variants in breast cancer and control group.

Genotype	BC Group N = 254 (100%)	Control Group N = 251 (100%)	OR (95% C.I)	*p* Value
***H19* rs3741219 T>C**				
T/T	67 (26.6)	100 (39.8)	1.00 (Reference)	
T/C	132 (52.0)	117 (46.6)	1.68 (1.13–2.50)	0.013
C/C	55 (21.7)	34 (13.5)	**2.41 (1.42–4.09)**	**0.001**
T/C + C/C vs. T/T	187 (73.6)	151 (60.2)	**1.84 (1.26–2.69)**	**0.001**
Allele				
T	266 (52.4)	317 (63.1)	1.00 (Reference)	
C	242 (47.6)	185 (36.9)	**1.55 (1.21–2.00)**	**0.001**
***POLR2E* rs3787016 T>C**				
T/T	79 (31.1)	103 (41.0)	1.00 (Reference)	
T/C	127 (50.0)	118 (47.0)	1.40 (0.95–2.06)	0.103
C/C	48 (18.9)	30 (12.0)	2.08 (1.21–3.58)	0.019
T/C + C/C vs. T/T	175 (68.9)	148 (59.0)	1.54 (1.06–2.22)	0.025
Allele				
T	285 (56.1)	324 (64.5)	1.00 (Reference)	
C	223 (43.9)	178 (35.5)	**1.42 (1.10–1.83)**	**0.007**
***MEG3* rs7158663 G>A**				
G/G	89 (35.0)	102 (40.6)	1.00 (Reference)	
G/A	125 (49.2)	118 (47.0)	1.21 (0.83–1.77)	0.365
A/A	40 (15.7)	31 (12.4)	1.47 (0.85–2.55)	0.206
G/A + A/A vs. G/G	165 (65.0)	149 (59.4)	1.26 (0.88–1.81)	0.228
Allele				
G	303 (59.6)	322 (64.1)	1.00 (Reference)	
A	205 (40.4)	180 (35.9)	1.21 (0.93–1.56)	0.159
***ANRIL* rs10757274 A>G**				
A/A	97 (38.2)	102 (40.6)	1.00 (Reference)	
A/G	121 (47.6)	117 (46.6)	1.08 (0.74–1.58)	0.733
G/G	36 (14.2)	32 (12.7)	1.18 (0.68–2.05)	0.647
A/G + G/G vs. A/A	157 (61.8)	149 (59.4)	1.10 (0.77–1.58)	0.637
Allele				
A	315 (62.0)	321 (63.9)	1.00 (Reference)	
G	193 (38.0)	181 (36.1)	1.08 (0.84–1.40)	0.567

Bold text highlights a statistically significant finding. *p* values were adjusted by Bonferroni test (0.012).

**Table 3:** Association of the *H19* rs3741219 T>C variant with demographic and clinical variables.

*H19* rs3741219 T>C						
Breast Cancer/Control				OR (95% CI); *p* Value		
Variable	TT	TC	CC	TC Versus TT	CC Versus TT	TC + CC Versus TT
**Age (years)**						
<50	42/74	57/78	31/19	1.28 (0.77–2.14); 0.039	**2.87 (1.44–5.70); 0.003**	1.59 (0.99–2.57); 0.069
>50	25/26	75/39	24/15	2.00 (1.02–3.91); 0.062	1.66 (0.71–3.88); 0.332	1.90 (1.00–3.62); 0.068
**Smoking status**						
**Yes**	17/16	31/14	18/5	2.08 (0.82–5.28); 0.185	3.38 (1.01–11.28); 0.079	2.42 (1.02–5.75); 0.069
**Drinking status**						
**Yes**	6/14	17/13	11/5	3.05 (0.92–10.11); 0.117	5.13 (1.23–21.35); 0.047	2.04 (0.69–6.02); 0.296
**TNM stage**						
I + II	21/100	43/117	24/34	1.75 (0.97–3.14); 0.081	**3.36 (1.66–6.79); 0.001**	**2.01 (1.15–3.51); 0.012**
III + IV	46/100	89/117	31/34	1.65 (1.05–2.58); 0.034	1.98 (1.08–3.60); 0.035	1.72 (1.13–2.63); 0.014
**Histologic type**						
Ductal	52/100	119/117	51/34	**1.95 (1.28–2.98); 0.002**	**2.88 (1.66–4.99); <0.001**	**2.16 (1.45–3.23); <0.001**
Lobular	14/100	11/117	4/34	0.67 (0.29–1.54); 0.465	0.84 (0.25–2.72); 1.000	1.18 (0.58–2.38); 0.769
Mixed	1/100	2/117	0/34	-----	-----	-----
**Histologic molecular subtype**						
Luminal A	39/100	74/117	30/34	1.62 (1.01–2.59); 0.057	2.26 (1.22–4.18); 0.013	1.76 (1.13–2.75); 0.016
Luminal B	12/100	33/117	13/34	2.35 (1.15–4.79); 0.025	3.18 (1.32–7.64); 0.014	**2.53 (1.28–5.20); 0.009**
Her2	12/100	13/117	8/34	0.92 (0.40–2.12); 1.000	1.96 (0.73–5.20); 0.270	1.15 (0.54–2.46); 0.845
Triple Negative	4/100	12/117	4/34	2.56 (0.80–8.20); 0.168	2.94 (0.69–12.40); 0.263	2.64 (0.86–8.15); 0.129

Bold text highlights statistically significant findings, ----- not applicable, *p* values were adjusted by Bonferroni test (0.012).

**Table 4:** Association of the *POLR2E* rs3787016 T>C variant with demographic and clinical variables.

*POLR2E* rs3787016 T>C						
_Breast Cancer/Control_				OR (95% CI); *p* Value		
Variable	TT	TC	CC	TC Versus TT	CC Versus TT	TC + CC Versus TT
**Age (years)**						
<50	44/69	70/82	16/20	1.33 (0.81–2.19); 0.302	1.25 (0.58–2.67); 0.695	1.32 (0.82–2.12); 0.301
>50	35/34	57/36	32/10	1.53 (0.81–2.88); 0.237	3.10 (1.32–7.29); 0.013	1.87 (1.04–3.39); 0.050
**Smoking status**						
**Yes**	17/16	33/14	16/5	2.21 (0.87–5.59); 0.142	3.01 (0.89–10.14); 0.126	2.42 (1.02–5.75); 0.069
**Drinking status**						
**Yes**	10/13	17/15	7/4	1.47 (0.50–4.32); 0.665	2.27 (0.51–9.98); 0.463	1.64 (0.59–4.55); 0.485
**TNM stage**						
I + II	27/103	42/118	19/30	1.35 (0.78–2.35); 0.341	2.41 (1.18–4.93); 0.023	1.57 (0.93–2.63); 0.111
III + IV	52/103	85/118	29/30	1.52 (0.92–2.20); 0.134	1.91 (1.04–3.52); 0.051	1.52 (1.00–2.30); 0.056
**Histologic type**						
Ductal	70/103	107/118	45/30	1.33 (0.89–1.99); 0.190	**2.20 (1.26–3.83); 0.007**	1.51 (1.03–2.20); 0.040
Lobular	9/103	17/118	3/30	1.64 (0.70–3.85); 0.340	1.14 (0.29–4.49); 1.000	1.54 (0.67–3.53); 0.400
Mixed	0/103	3/118	0/30	-----	-----	-----
**Histologic molecular subtype**						
Luminal A	48/103	67/118	28/30	1.21 (0.77–1.92); 0.462	2.00 (1.07–3.71); 0.039	1.37 (0.89–2.11); 0.174
Luminal B	16/103	31/118	11/30	1.69 (0.87–3.26); 0.157	2.36 (0.99–5.62); 0.083	1.82 (0.97–3.42); 0.080
Her2	11/103	17/118	5/30	1.34 (0.60–3.01); 0.595	1.56 (0.50–4.84); 0.643	1.39 (0.64–2.99); 0.509
Triple Negative	4/103	12/118	4/30	2.61 (0.81–8.37); 0.156	3.43 (0.80–14.55); 0.181	2.78 (0.90–8.56); 0.106

Bold text highlights statistically significant findings, ----- not applicable, *p* values were adjusted by Bonferroni test (0.012).

**Table 5:** Association of the *MEG3* rs7158663 G>A variant with demographic and clinical variables.

*MEG3* rs7158663 G>A						
Breast Cancer/Control				OR (95% CI); *p* Value		
Variable	GG	GA	AA	GA Versus GG	AA Versus GG	GA + AA Versus GG
**Age (years)**						
<50	48/69	58/83	24/19	1.00 (0.61–1.65); 1.000	1.81 (0.89–3.67); 0.136	1.15 (0.72–1.84); 0.627
>50	41/33	67/35	16/12	1.54 (0.83–2.84); 0.220	1.07 (0.44–2.58); 1.000	1.42 (0.79–2.54); 0.299
**Smoking status**						
**Yes**	24/13	34/18	8/3	1.02 (0.42–2.47); 1.000	1.44 (0.32–6.40); 0.903	1.08 (0.46–2.54); 1.000
**Drinking status**						
**Yes**	8/12	21/17	5/3	1.85 (0.61–5.56); 0.407	2.5 (0.46–13.52); 0.509	1.95 (0.67–5.67); 0.333
**TNM stage**						
I + II	31/102	44/118	13/31	1.22 (0.72–2.08); 0.534	1.37 (0.64–2.95); 0.529	1.25 (0.75–2.08); 0.442
III + IV	58/102	81/118	27/31	1.20 (0.78–1.85); 0.452	1.53 (0.83–2.81); 0.169	1.27 (0.84–1.91); 0.285
**Histologic type**						
Ductal	77/102	113/118	32/31	1.26 (0.85–1.87); 0.276	1.36 (0.76–2.43); 0.357	1.28 (0.88–1.87); 0.216
Lobular	10/102	11/118	8/31	0.95 (0.38–2.39); 1.000	2.63 (0.95–7.24); 0.101	1.30 (0.58–2.91); 0.659
Mixed	2/102	1/118	0/31	-----	-----	-----
**Histologic molecular subtype**						
Luminal A	57/102	61/118	25/31	0.92 (0.59–1.44); 0.820	1.44 (0.77–2.67); 0.314	1.03 (0.67–1.57); 0.964
Luminal B	14/102	37/118	7/31	2.28 (1.16–4.46); 0.021	1.64 (0.62–4.43); 0.472	2.15 (1.12–4.12); 0.028
Her2	12/102	16/118	5/31	1.15 (0.52–2.54); 0.881	1.37 (0.44–4.19); 0.800	1.19 (0.56–2.54); 0.777
Triple Negative	6/102	11/118	3/31	1.58 (0.56–4.43); 0.528	1.64 (0.38–6.96); 0.181	1.59 (0.59–4.29); 0.485

Bold text highlights statistically significant findings, ----- not applicable, *p* values were adjusted by Bonferroni test (0.012).

**Table 6:** Association of the *ANRIL* rs10757274 A>G variant with demographic and clinical variables.

*ANRIL* rs10757274 A>G						
Breast Cancer/Control				OR (95% CI); *p* Value		
Variable	AA	AG	GG	AG Versus AA	GG Versus AA	AG + GG Versus AA
**Age (years)**						
<50	47/74	61/72	22/25	1.33 (0.80–2.19); 0.315	1.38 (0.70–2.73); 0.442	1.34 (0.84–2.15); 0.258
>50	50/28	60/45	14/7	0.74 (0.40–1.36); 0.424	1.12 (0.40–3.10); 1.000	0.79 (0.44–1.42); 0.537
**Smoking status**						
**Yes**	24/14	30/17	12/4	1.02 (0.42–2.50); 1.000	1.75 (0.47–6.48); 0.598	1.16 (0.50–2.70); 0.886
**Drinking status**						
**Yes**	17/13	13/17	4/2	0.58 (0.21–1.62); 0.438	1.52 (0.24–9.67); 1.000	0.68 (0.25–1.81); 0.605
**TNM stage**						
I + II	31/102	45/117	12/32	1.26 (0.74–2.14); 0.459	1.23 (0.56–2.68); 0.742	1.25 (0.75–2.08); 0.442
III + IV	66/102	76/117	24/32	1.00 (0.65–1.53); 1.000	1.15 (0.62–2.14); 0.752	1.03 (0.69–1.54); 0.938
**Histologic type**						
Ductal	84/102	104/117	34/32	1.07 (072–1.59); 0.777	1.29 (0.73–2.26); 0.456	1.12 (0.77–1.62); 0.597
Lobular	13/102	14/117	2/32	0.93 (0.42–2.09); 1.000	0.49 (0.10–2.28); 0.549	0.89 (0.38–182); 0.814
Mixed	0/102	3/117	0/32	-----	-----	-----
**Histologic molecular subtype**						
Luminal A	57/102	66/117	20/32	1.00 (0.64–1.57); 1.000	1.11 (0.58–2.13); 0.862	1.03 (0.67–1.57); 0.964
Luminal B	18/102	30/117	10/32	1.45 (0.76–2.76); 0.324	1.77 (0.74–4.22); 0.288	1.52 (0.82–2.80); 0.228
Her2	16/102	13/117	4/32	1.40 (0.63–3.01); 0.503	0.79 (0.24–2.55); 0.920	0.72 (0.35–1.50); 0.501
Triple Negative	6/102	12/117	2/32	3.45 (1.26–9.44); 0.021	1.06 (0.20–5.52); 1.000	1.59 (0.59–4.29); 0.485

Bold text highlights statistically significant findings, ----- not applicable, *p* values were adjusted by Bonferroni test (0.012).

**Table 7:** Logistic regression analysis for the *H19* and *POLR2E* variants analyzed with variables associated.

Independent Variable	Regression Coefficient	Standard Error	Wald Test	Degrees of Freedom	*p* Value	OR (95% IC)
**Age** **>50 vs. <50**	0.641	0.191	11.250	1	**0.001**	**1.89 (1.30−2.76)**
**Smoking status** **Yes vs. No**	0.905	0.260	12.115	1	**0.001**	**2.47 (1.48−4.11)**
**Drinking status** **Yes vs. No**	–0.449	0.303	2.187	1	0.139	0.63 (0.35–1.15)
***H19* rs3741219** **TC + CC**	0.577	0.200	8.278	1	**0.004**	**1.78 (1.20−2.63)**
***POLR2E* rs3787016** **TC + CC**	0.420	0.194	4.682	1	0.030	1.52 (1.04–2.22)
**Constant**	–1.191	0.327	13.227	1	0.304	
**Model**	X^2^ = 42.516 d.f. = 7 ***p* =** <**0.001**					

Bold values indicate statistically significant findings. X^2^ = chi square, d.f = degrees of freedom. *p* values were adjusted by Bonferroni test (0.012).

**Table 8:** Regulatory targets of *H19* and its associated ceRNAs and miRNAs.

Regulatory Element	Type	Interacting Molecule	Associated Cellular Process	Functional Role
*ESR1*	lncRNA	*H19*	Chemoresistance	Paclitaxel resistance
*H19*	Protein interaction	*EZH2*	Chemoresistance	Paclitaxel resistance
*H19*	Histone modification	*PMAIP1*	Apoptosis regulation	Pro-apoptotic modulation
*HOTAIR/91H*	lncRNA/Transcription factor	*H19*	Transcriptional regulation	Upstream regulators of *H19*
*E2F1*	Transcription factor	*H19*	Cell proliferation	Upstream regulators of *H19*
*H19*	miRNA Sponge (miR-200 b/c)	*CYTH3*	Epithelial–Mesenchymal Transition (EMT)	EMT inhibition
*H19*	ceRNA (let-7 family)	*LIN28A*	Stemness differentiation	Repression of pluripotency factors
*H19*	miRNA host (miR-675-5p)	*CBL*	Cell proliferation	Regulation of growth signaling
*H19*	miRNA Sponge (miR-152)	*DNMT1*	Epigenetic regulation	DNA methylation control

lcnRNA = long non-coding RNAs, miRNA = microRNA, ceRNA = competing endogenous RNA, EMT = Epithelial–Mesenchymal Transition.

## Data Availability

The original contributions presented in this study are included in the article and its Supplementary Material. Further inquiries can be directed to the corresponding author.

## Supplementary Materials

The following supporting information can be downloaded at: https://www.mdpi.com/article/10.3390/ncrna12030019/s1, Figure S1: Transcription factor binding evidence at the *POLR2E* locus identified by RegulomeDB; Table S1: Immunohistochemical expression of *RBFOX2* and *AGO2* in breast cancer according to histological subtype.

## Abbreviations

The following abbreviations are used in this manuscript:

AGO2	Argonaute-2
*ANRIL*	Antisense Non-Coding RNA in the INK4 Locus
ANOVA	Analysis of variance
BC	Breast cancer
BMI	Body Mass Index
bp	Base pairs
BRCA	Breast cancer dataset (TCGA)
CADD	Combined Annotation Dependent Depletion
ceRNA	Competing endogenous RNA
ChIP-seq	Chromatin immunoprecipitation sequencing
CI	Confidence interval
DC	Ductal carcinoma
DNA	Deoxyribonucleic acid
dNTPs	Deoxyribonucleotide triphosphates
EMT	Epithelial–Mesenchymal Transition
ER	Estrogen receptor
ERα	Estrogen receptor alpha
ESMO	European Society for Medical Oncology
GEPIA2	Gene Expression Profiling Interactive Analysis 2
GERP	Genomic Evolutionary Rate Profiling
GTEx	Genotype-Tissue Expression
GWAS	Genome-wide association studies
HR	Hazard ratio
HWE	Hardy–Weinberg equilibrium
IGF2	Insulin-like growth factor 2
IHC	Immunohistochemistry
IMSS	Instituto Mexicano del Seguro Social
kb	Kilobases
LB	Lobular carcinoma
lncRNA	Long non-coding RNA
*MEG3*	Maternally Expressed Gene 3
MET	Mesenchymal–Epithelial Transition
miRNA	MicroRNA
mRNA	Messenger RNA
NCCN	National Comprehensive Cancer Network
NICE	National Institute for Health and Care Excellence
nt	Nucleotides
OR	Odds ratio
PCC	Pearson correlation coefficient
PCR	Polymerase chain reaction
PCR-RFLP	Polymerase chain reaction-restriction fragment length polymorphism
PHRED	Phred-scaled deleteriousness score (CADD)
*POLR2E*	RNA Polymerase II subunit E
PR	Progesterone receptor
RBFOX2	RNA-binding fox-1 homolog 2
RFLP	Restriction fragment length polymorphism
RNA	Ribonucleic acid
RNA-seq	RNA sequencing
SD	Standard deviation
SNP	Single-nucleotide polymorphism
SNV	Single-nucleotide variant
SPSS	Statistical Package for the Social Sciences
TCGA	The Cancer Genome Atlas
TFBS	Transcription factor binding site
TNM	Tumor–node–metastasis
TPM	Transcripts per million
