# Supplementary material for: Investigation of Long Non-Coding RNAs H19 rs3741219, MEG3 rs7158663, POLR2E rs3787016, and ANRIL rs10757274 with Breast Cancer Susceptibility and Clinicopathological Characteristics in a Mexican Population
Source: Noncoding RNA. 2026 Jun 4;12(3):19. doi: 10.3390/ncrna12030019 (PMC13305036; doi:10.3390/ncrna12030019)
Supplement: Supplementary file 1 [file ncrna-12-00019-s001.zip › Supplementary Table S1.pdf]

Table S1. Immunohistochemical expression of RBFOX2 and AGO2 in breast cancer according to histological subtype.

Protein	Positive cases/Total	% Positive	High Intensity (Histologic Types)	Medium Intensity (Histologic Types)	Low Intensity (Histologic Types)
RBFOX2	8/12	67%	0%	0%	8 (67%)<br>7 lb/1dc
AGO2	12/12	100%	3 (25%)<br>2 lb/1dc	8 (67%)<br>1lb/7dc	1 (8%)<br>1lb
POLR2A*	N/A	--	--	--	--

*IHC data for POLR2A was not available in The Human Protein Atlas at the time of analysis. LB = lobular carcinoma; DC = ductal carcinoma.
