# Supplementary figures and images for: Investigation of Long Non-Coding RNAs H19 rs3741219, MEG3 rs7158663, POLR2E rs3787016, and ANRIL rs10757274 with Breast Cancer Susceptibility and Clinicopathological Characteristics in a Mexican Population
Source: Noncoding RNA. 2026 Jun 4;12(3):19. doi: 10.3390/ncrna12030019 (PMC13305036; doi:10.3390/ncrna12030019)

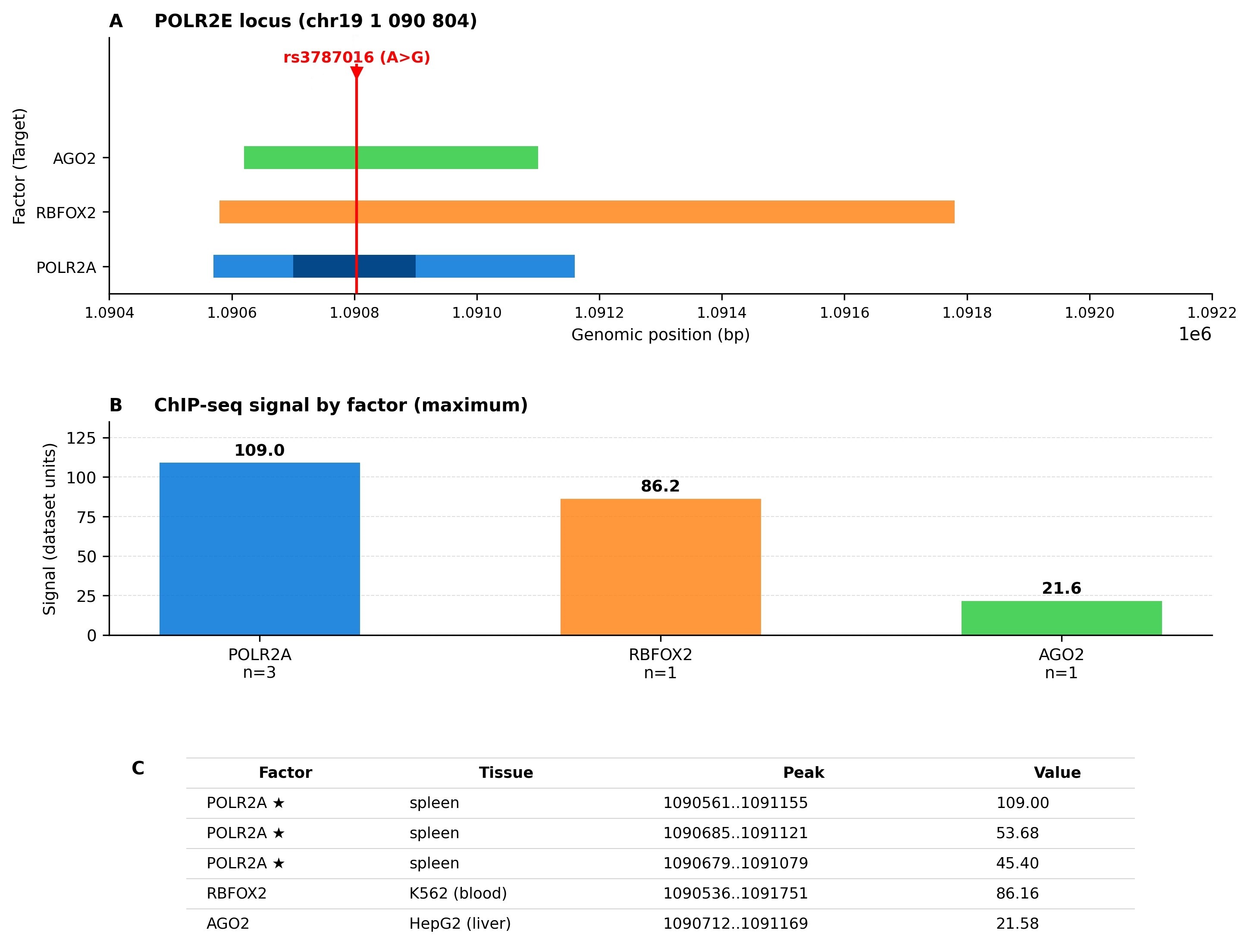

Supplement: Supplementary file 1 [file ncrna-12-00019-s001.zip › FigureS1_300dpi_3.jpg]
